# Viewpoint changes of medical sciences graduates in evaluating the performance of faculty members: a qualitative study

**DOI:** 10.1186/s12909-022-03238-5

**Published:** 2022-03-18

**Authors:** Hanieh Azizi, Mozhgan Beheshid, Kamal Gholipour, Saeed Aslan-Abadi, Ali Azadifar, Mahasti Alizadeh

**Affiliations:** 1 Medical Education Research Center, Student Research Committee, Tabriz University of Medical Sciences, Tabriz, Iran; 2 Medical Education Research Center, Health Management and Safety Promotion Research Institute, Tabriz University of Medical Sciences, Tabriz, Iran; 3 Tabriz Health Services Management Research Center, Tabriz University of Medical Sciences, Tabriz, Iran; 4 Medical Education Research Center, Faculty of Medicine, Health Management and Safety Promotion Research Institute, Tabriz University of Medical Sciences, Tabriz, Iran; 5 Department of Radiology, Tabriz University of Medical Sciences, Tabriz, Iran; 6 Social Determinants of Health Research Center, Health Management and Safety Promotion Research Institute, Tabriz University of Medical Sciences, University Rd, Tabriz, Iran

**Keywords:** Students, Faculty, University professor, Attitude of health personnel, Academic development, Faculty evaluation, Qualitative research, Iran

## Abstract

**Background:**

This study aimed to identify which dimensions of faculty members’ evaluation criteria changed from the viewpoint of students after their graduation, and to determine the effective factors in changing their viewpoints.

**Methods:**

This study was carried out through the qualitative approach and with conventional content analysis method. The target population included all graduates who accomplished their job duty and had a working experience of 2-4 years. A purposive sampling technique with maximum variation used to recruit and interview. Twenty-eight in depth semi-structured interviews were conducted in Tabriz University of Medical Sciences (TBZMED), Iran. The data were analyzed using content analysis.

**Results:**

The data analysis led to the development of two themes and 8 categories. The two types of changes in the viewpoint that were experienced by graduates in evaluating the performance of faculty members were: individual and environmental. Individual factors included the responsibility of graduates, social maturity, personal experience, intellectual maturity, understanding the causes of teachers’ behaviors, and understanding the importance of evaluation. The environmental factors were applicability of learning experiences in the work environment and workplace conditions.

**Conclusion:**

From the perspective of graduates, the importance of some evaluation criteria in the educational, professional, and personal dimensions changed over their study period due to some factors, such as personal experience, experiences in the work environment, workplace conditions, and intellectual maturity.

## Introduction

Evaluation of the performance of faculty members is one of the important tools in the educational processes, which makes it possible to identify the strengths and weaknesses to take an effective step through improving the positive aspects in removing the shortcomings. One of the problems of universities and educational centers is the evaluation of the faculties’ activities [1, 2]. There are various models available for evaluation of faculty members, including evaluation by the authorities, peer groups, students, self-evaluation, evaluation of students’ learning, and evaluation of the educational materials content [3]. Evaluation of faculty members is one of the important ways to ensure faculty’s academic achievements and promote faculty’s academic development, although some faculties and learners are dissatisfied with the evaluation process [4, 5]. Some researchers have identified the faculty evaluation by students as the best way because they believe that students are the only ones directly trained by the faculty. In contrast, another group believes that students are not mature enough to evaluate faculty members appropriately because they are not familiar with the concept of teaching and learning and they might simply be deluded by an attractive show or a good score [6].

In higher education universities including medical sciences universities, teaching by faculty members is considered a qualitative index of education, which is evaluated by different methods and by different sources including students, administrators, peers, and self-evaluation [7]. The continuing professional development of faculties is also one of the key issues in higher education, and faculty evaluation can provide important information related to promotion, intervention, competence, or personal development and growth [8].

Evaluation is one of the most important bases for improving the quality of education and is referred to as a systematic process for collecting, analyzing, and interpreting information [9, 10]. Today, evaluation of faculties’ educational activities is carried out through more than thousands of different types of evaluation questionnaires [11]. The purpose of the faculties’ evaluation is to improve the process of teaching and its effectiveness, which is carried out in a variety of ways, such as evaluation by students (the most common method), colleagues, department heads, and review of educational records [12]. However, a valid evaluation of the faculty members’ performance can be done by graduates. In most of the universities graduates’ views about their teachers are not taken years after graduation and since few studies have been conducted on the change in graduates’ viewpoints toward evaluation criteria of faculty members compared to their studying period, this study aimed to identify which dimensions to evaluate the performance of faculty members changed from the viewpoint of graduates compared to their studying period. It was also attempted to determine the effective factors changing their viewpoints.

## Methods

This study was carried out through the qualitative approach and with conventional content analysis method. The target population included all graduates of Tabriz University of medical sciences at North-West of Iran. According to their rich experience or expertise in evaluating the performance of faculty members and their willingness to participate in the study, interviewees were selected by purposive sampling. Participants were graduates who accomplished their job duty and had a working experience of 2-4 years In order to achieve maximum variation in the samples, graduates were enrolled in the study in a spectrum of weak to strong in terms of mean scores, extracurricular activities, and research. Semi structured interviews, focused group discussion and field notes were used to gather data. Thirty-one Face to face in-depth semi structured interviews provided deep and rich level of understanding about the phenomena that covering the changing viewpoint experiences of graduates. The researcher introduced herself and expressed aim and method of study before obtaining the informed consent of participants. The interviews conducted at participants work places and began with a opening question about participants’ experiences of academic members’ performance evaluation and then probing questions were conducted according to participants’ answers. Each interview lasted for 30-60 min and was tape recorded and transcribed verbatim. Although participants were encouraged to give more data and to discuss their experiences during the interviews, researchers conducted a re-interview if there were any ambiguities in the participants’ statements (3 cases). Also observation and field notes was used for gathering data.

Participants were 28 graduates of Tabriz University of medical sciences and 3 experts in medical education. Also 1 focused group discussion was held and 4 experts working in the field of evaluation of faculty members’ performance as well as 6 faculty members with the highest and lowest grades of evaluation were included. Sampling and data coding continued till data saturation when no new code was obtained during interviews and repetition of the previous categories and codes. Interviews were conducted by HA who was MSc in medical education. MB, KG, SA and MA were faculty members who reviewed the interviews. The interviewer explained the goals of the study to the interviewees. The facilitator in the FGD sessions were a professor of community medicine (MA), who had a good research and work experience in the field of faculty members evaluation. The time and place of the interviews were determined according to the interviewees’ preferences and most of them were in their workplace no one else was in the interview except interviewer and the participant. Interviews were recorded by recorders after getting permission from the participants then transcribed word by word. Transcripts returned to participants for comment and/or correction each interview lasted for 30-60 min. The demographic information of participants was collected and an informed consent was obtained from all participants.,

Data were analyzed using content analysis. Data analysis started from the first interview along with the subsequent interviews (concurrent analysis); so that the notes were studied several times in order to obtain a comprehensive understanding of the text. Then, the text was read line by line. After providing the necessary training and defining the concepts to the encoders, two encoders extracted the concepts (HA MB and KG). It was attempted to combine similar codes to increase the coders’ agreement on the coding and to easily distinguish the codes.

Trustworthiness of study method and procedure were ensured by a variety of strategies such as member checking, audit trail, prolonged engagement, purposive sampling and peer debriefing. We used researchers’ immersion in data using prolonged involvement and close communication with participants to reach credibility. We used purposive sampling from a vast range of participants from different disciplines and experiences to improve transferability of results. Additionally, we used audit trail by two academic experts to check the data collection and analysis procedures as well as compliance of the study with the research protocol. We also sent the results and primary data to the participants for their approval.

The method and procedures of the research project was reviewed and approved by the Research Ethics Committee of the university. All stages of the study, including data collection, storage, analysis, and reporting were kept confidential and the data were available only to the members of the research team.

## Results

In this study, semi-structured interviews were carried out with 28 graduates, of whom 46% were males and 54% were females. Most of the participants were working in hospitals. Employment status of the participants was as follows: private sector: 21%, hospitals: 43%, administrative units: 18% and health centers: 18% (Table 1).

**Table 1:** Demographic Characteristics of the study Participants

Characteristics	No	Percent (%)
**Gender**		
Male	13	46
Female	15	54
**Degree**		
BSc	10	35
MSc	9	32
General practitioner	5	19
Specialist	2	7
PhD	2	7
**Workplace**		
Hospital	12	43
Private sector	6	21
Administrative units	5	18
Health Center	5	18

**Fig. 1:**
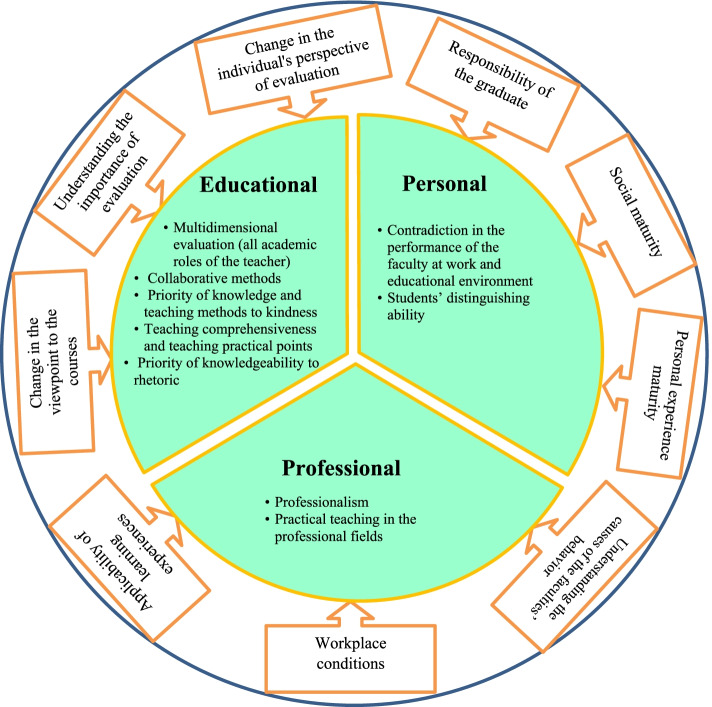
Dimensions and Factors Affecting the Graduates’ Viewpoints in Evaluating the Performance of Faculty Members

Based on the viewpoint of the participants, the dimensions of change in the viewpoints of graduates in evaluating the performance of faculty members were categorized into three main groups including educational, professional, and individual ones. Also, factors affecting the change were divided into two categories: individual factors and environmental factors (Fig. 1).

### Educational dimensions

Educational dimensions affecting the evaluation of the faculty from the graduates’ viewpoints included five subcategories: multidimensional evaluation (all academic roles of a teacher), collaborative methods, importance of knowledge and teaching methods, teaching comprehensiveness, teaching practical tips, and priority of knowledgeability to rhetoric.

Multidimensional evaluation (all academic roles of the teacher) is considered as one of the subcategories of educational dimensions. This indicator illustrates that much attention should be paid to all dimensions of the teacher’s role in the evaluation.

In this regard, a nurse holding a master’s degree and working in the hospital said: “The faculty who has taught one-dimensional science to the students will be scored lower in the evaluation by the graduates” (P 15).

Also, an environmental health expert working in the health center strongly believed in the importance of paying attention to all the roles of the faculties: “After the graduation, people score the same after graduation for the faculties who were good both ethically and at teaching” (P 21).

Collaborative method was identified as another category influencing the educational dimensions. Although assigning research and interactive teaching methods were not accepted at the time of studying, the graduates believed that their importance was clearly visible after graduation.

In this regard, the environmental health expert working at the health center indicated that: “After graduation, students appreciate those faculties who motivate and make students conduct research” (P 21).

The importance of knowledge and teaching methods is another subcategory influencing educational dimensions. This issue underlines the fact that the faculties who were gentle and kind but did not teach well were rated low by the graduates; however, the strict ones who taught well received a high rating.

A dentist in the private sector said that: “Kind and easy-going teachers are graded lower by the graduates” (P 27).

Teaching practical points is another subcategory influencing the change of graduates’ viewpoint in evaluating the educational performance of faculty members. The graduates emphasized that faculties should teach a series of applied subjects during their courses (emphasis on practical training).

In this regard, a radiology technician working for the private sector said: “The faculties who work practically with the students and help them learn much better than their colleagues are graded higher” (P 18).

Also, a senior radiologist at hospital expressed that: “After graduation, the radiology graduates find out that they have not been trained appropriately (not practically), and the result is poor evaluation of the faculties” (P 11).

The priority of knowledgeability to rhetoric is another subcategory influencing the educational dimensions. Most graduates prioritized knowledgeability to rhetoric skills.

A nutrition expert working in a public hospital indicated that: “The faculties who enjoy rhetoric skills but do not teach well are rated lower by the graduates” (P 24). However, according to the viewpoints of participants in this study, the importance of having good rhetoric and teaching skills is higher in comparison with kindness. In this respect, a senior health services management graduate working for the public sector expressed that: “Verbal competence and holding efficient training sessions is important to the graduates after entering job market” (P 10).

In this study, according to the participants’ viewpoints, the main priorities for the graduates compared to their studying period included: having good academic knowledge, enjoying rhetoric skills and good verbal competence, and being gentle and kind.

#### Professional dimensions

Professional dimensions affecting the evaluation of faculties from the viewpoint of graduates were classified into two subcategories: professionalism and the applicability of teaching in the professional field.

Professionalism is considered as a professional dimension. For example, a senior nursing graduate working in a public hospital stated that: “Graduates pay more attention to factors such as professionalism in faculties after entering job market” (P 15). Also, a laboratory science staff at a public hospital indicated that: “The faculties in whom the graduates find no proficiency are rated lower after entering the job market” (P 22).

The applicability of teaching in the professional field was identified as another subcategory influencing the professional dimensions. This component confirms that the faculties who use the applied method in teaching are rated higher after the students’ graduation compared to the university years.

A psychiatrist working in a public hospital said that: “The difference in the attitude of the graduates compared to studying period was in the applicability of taught materials” (P 19). Also, an anesthetic technician working in the hospital indicated that: “The faculties who get the students to work more practically are rated well after graduation” (P 23).

On the other hand, a number of graduates believed that the weakness of the educational system as well as curriculum makes it possible for the students to learn key and applied points after graduation with trial and error, either alone or by contextual obligation. In this regard, a general practitioner at the health center believed that: “Due to inefficiency of the educational system, the graduates are not informed enough about common diseases” (P 13). He also maintained that: “After graduation, the individual learns key and vital points practically” (P 13).

#### Personal dimensions

The personal dimensions affecting the evaluation of the faculties from the viewpoint of graduates were classified into three subcategories: contradiction in the performance of the faculties in the workplace, educational environment, and the distinguishing ability of students.

Contradiction in the performance of the faculty in the work and educational environment is considered as one of the subcategories of the personal dimensions. The graduate, as a colleague of faculty member in the workplace, comes to recognize a series of facts that differ from his/her studies. A laboratory science expert working at an educational hospital indicated that: “The work environment and working with the faculties as colleagues results in a lower rating of the faculty” (P 22).

Students’ distinguishing ability was the second subcategory of the personal dimensions. Based on the graduates’ view, the individuals’ distinguishing ability in the faculty evaluation partially depends on the underachiever or overachiever students. As a result, most students believe that smart students usually fill the evaluation forms more accurately and they do not change their evaluation after graduation. In this regard, a general practitioner at the health center said that: “An overachiever’s attitude does not change after graduation” (P 13). Meanwhile, the same graduate believed that: “Major changes are usually seen in an underachiever’s attitude after entering the job market” (P 13).

### Personal factors affecting the change of graduates’ viewpoints

Personal factors influencing the faculties’ evaluation from the viewpoint of graduates included seven subcategories: graduates’ responsibilities, social maturity, personal experience and intellectual maturity, change in the individual perspective regarding evaluation, change in viewpoint about the courses, understanding the causes of the faculties’ behavior, and understanding the importance of evaluation.

Improving the graduates’ responsibilities is considered as one of the subcategories. An individual must be taught to be responsible after graduation, so that a general practitioner at the health center indicated that: “The graduates have no sense of responsibility due to inappropriate educational system that does not promote responsibility” (P 13). Also, the lack of responsibility during the studying period causes the student to regret his/her incorrect evaluation. In this regard, a nurse working in a hospital mentioned that: “The graduates regret their incorrect evaluation after graduation” (P 3).

Social maturity is another subcategory. Such factors as being married, having a child, and other social components can play a role in changing the graduates’ viewpoints. A senior graduate of medical information at the nursing school indicated that: “I appreciated the faculty’s teaching in a better way after graduating and becoming a parent” (P 2).

Personal experience and intellectual maturity is considered as a subcategory affecting the change. A PhD graduate of pharmacology working in the private sector mentioned that: “Postgraduate students have made more documented evaluations due to older age and higher experience” (P 16). A senior medical librarian working at the research and technology department also mentioned that: “The effect of intellectual maturity and the passage of time affect the evaluation of faculties.” (P 5).

The change in the viewpoints of individuals regarding faculty members’ evaluation criteria is the most important subcategory. Participants in this subcategory mentioned the role of such factors as: passage of time, change in educational level, and labor market.

A senior medical information graduate working at the research and technology department indicated that: “The change of graduates’ viewpoint about the functional role of the teachers usually happens at the end of the undergraduate level” (P 1).

However, some other participants did not believe in any kind of change in the viewpoints after graduation. A general dentist working in a private clinic mentioned that: “After graduation no significant modification was observed in the answers provided to the evaluation questions” (P 7). The considerable point in the category of personal viewpoint modification is that the individual’s viewpoint and evaluation scores usually change regarding ethics after graduation, but they do not alter concerning education. As a laboratory science staff working in a hospital he expressed that: “The education score remains unchanged after the graduation” (P 22). Further in his interview, he indicated that: “The individuals do not change the scores related to the faculty’s performance after graduation” (P 22). Also, a senior physiotherapist working at a clinic affiliated to the medical university reflected that: “Thinking back, hardworking students do not change their evaluation scores” (P 25).

The participants also mentioned that the work environment and its requirements would change their view about the courses they passed. In other words, the more practical the courses have been in the work environment, the more important the courses and the teachers have become. A PhD graduate of pharmaceutical medicine said that: “Graduates acknowledge the significance of academic courses when entering the job market” (P 16).

Understanding the causes of the faculty’s behavior is another subcategory affecting change of graduates’ viewpoint on the faculty evaluation. In this subcategory, the participants stated that after graduation and entering the job market, they appreciated the faculty’s strict and serious behaviors. A nurse working in a hospital specified that: “An understanding of the effectiveness of the faculty’s performance after entering the job market is really important” (P 4). In addition, a general practitioner working in a health center expressed “a better understanding of work, behavior, and performance of teachers after entering the job market” (P 6).

Understanding the importance of evaluating is another subcategory affecting the change in graduates’ viewpoint. Participants acknowledged that after graduation and entering the job market, they realized the importance of evaluation.

n this regard, a nurse working at a hospital emphasized “the change in attitude, and consequently, in the perspective about evaluation” (P 4). A number of graduates declared that they would fill out the evaluation forms carefully since they had not received any feedback during their studies. However, a senior immunologist at the hospital reflected that: “The students do not fill the evaluation sheets carefully since they do not get any feedback during their studies” (P 12).

#### Environmental factors

Environmental factors were categorized into two subcategories of applicability of learning experiences in the work environment and workplace conditions.

The applicability of learning experiences in the work environment is a subcategory of environmental factors affecting change of graduates’ viewpoint. Regarding this component, the graduates only remember those faculties whose teachings were useful and practical at the workplace after graduation.

A nutrition consultant at a health center said that: “Only after graduation, the individual will find out which courses and teachers were useful and good” (P 24). A PhD graduate in pharmaceutics working at the private sector believed that: “After graduation and starting a career, the students look at applied courses more obsessively” (P 16). A remarkable point made by most graduates is that a strict teacher with great teaching skills is preferred by students after their graduation. For instance, a nutrition consultant at a health center indicated that: “One refers to the handout of a good teacher even after graduation” (P 24).

The workplace condition is the second subcategory of environmental factors affecting the change of viewpoint. Based on the experiences of the graduates, this component indicates that the work environment and the workplace conditions, as well as starting a career, had been effective in changing the viewpoints of the graduates. A nurse in a hospital said that: “Starting a career and understanding its requirements change the viewpoint about the faculty, their teaching methods, and evaluation results” (P 3).

## Discussion

In this study, the change in the graduates’ viewpoints regarding the evaluation of the faculty members was categorized into viewpoint change dimensions and factors affecting the change. The viewpoint change dimensions on the evaluation of the faculties were categorized as educational, professional, and personal dimensions. Factors influencing the change were classified into two groups including individual factors and environmental ones.

Based on the participants in this study, the first dimension was educational that included five multidimensional evaluation subcategories (all academic roles of the faculties), collaborative methods, importance of skills and teaching methods, teaching practical points in education, and the priority of knowledgeability to rhetoric skills. In this regard, in a study carried out by Azer (2005), the qualities of a good teacher were identified in 12 domains, including interest in work, considering differences, respectful behavior, motivation of students and colleagues, ability to create a trusted educational environment, reinforcing critical thinking, encouraging creative work, focus on teamwork, paying attention to continuous promotion of educational skills, and giving positive feedback to students [13]. Moreover, a study by Grissom and Loeb (2017) included the knowledge of learners, executive planning, engagement and collaboration, educational evaluation, communication, professional principles, and the educational environment as areas for the evaluation of a university teacher. In addition, they indicated that promoting critical thinking, student motivation, reinforcement of individual skills and educational performance are recognized as the criteria for the effectiveness of the faculty in the workplace. Furthermore, establishing a working relationship, supporting colleagues, participating in executive and leadership affairs, and establishing social relationships have been mentioned as criteria for the effectiveness of the teacher in a non-classroom environment [14]. In a study by Das et al., the knowledge about subject and expertise was considered as educational dimensions of the faculty members [15]. In the present study, from the perspective of the participants, knowledgeability was superior to the rhetoric skills. However, in the study by Bergman et al., communication skills were identified as more important items [16]. Based on the study by Onwuegbuzie et al., being knowledgeable, professional, interested, facilitating, communicating, guiding, ethical, and responsive were identified as the categories for influential faculty in priority order [17]. In other words, similar to our study, their study showed the priority of the faculty’s knowledgeability to teaching activities and performance.

In this study, some of the dimensions of the change in the viewpoints such as the priority of knowledge and teaching methods to good behavior and the priority of knowledgeability to rhetoric skills were considered in relation to change in terms of the graduates’ viewpoints.

In the present study, the priority of knowledge and teaching method to behavioral traits was one of the educational dimensions in changing the viewpoints of graduates in evaluating the performance of faculty members. In this regard, based on the study by Shevlin et al., applied and clinical teaching was identified as one of the most important priorities in the evaluation of teachers due to the importance in the real environment for skill acquisition. Also, mastery of course was considered as the most important component of the faculty evaluation from the viewpoint of students and interns, though educational commitment in teaching and education was considered as the most important criterion of evaluation from the viewpoint of educational managers [18].

Based on the findings of the present study, the contradiction between the performance of the faculty in the work and education environments and the students’ distinguishing ability were classified as personal dimensions of the change in the viewpoint of graduates when evaluating the performance of teachers. In a study of Apodaca et al., the teacher’s ability and lesson characteristics were considered as the most important aspects of the faculties’ evaluation [19]. The main focus on students’ opinions and judgments is considered as the most important criterion in the faculties’ evaluation in most evaluation models [6]. On the other hand, some others considered these evaluations unreliable and invalid, and they believed that students are not mature enough to judge educational aspects and they are usually delighted with an attractive show or a good score [20]. However, in the present study, the students’ distinguishing ability was considered as one of the moderators in the accuracy of the faculty’s evaluation from the students’ viewpoints. Accordingly, in the current study, improving responsibility, social maturity, personal experience and intellectual maturity, change in the personal perspective regard evaluation, change in the viewpoints toward the courses, understanding the causes of the teachers’ behaviors, and understanding the importance of evaluating were identified as factors influencing the change in viewpoints of graduates when evaluating the performance of teachers.

According to the graduates’ viewpoints, the applicability of teaching in the professional field is one of the most important aspects of the faculties’ evaluation. In this regard, Emery et al. determined that although students’ evaluation about the effectiveness of the evaluation in educational effectiveness was more based on human relations and personality type of the faculties, the evaluation of educational effectiveness should be based on educational area [20]. Also, from the participants’ perspective, the applicability of learning experiences in the work environment and workplace condition was one of the factors that changed the view of the graduates compared to their studying period.

## Conclusion

Based on the results of this study, the viewpoint of graduates on the importance of some aspects of faculty members’ evaluation criteria in comparison to their studying period was changed in the educational, professional, and personal dimensions. This change was undertaken due to some personal factors, such as graduates’ responsibilities, social maturity, personal experience and intellectual maturity, change in the individual perspective regard evaluation, change in viewpoint about the courses, understanding the causes of the faculties’ behavior, and understanding the importance of evaluation by graduates. Also, environmental factors included applicability of learning experiences in the work environment and workplace conditions. After graduation, graduates state that faculty members who taught scientifically and rigorously in a practical way are better teachers than kind teachers who were not scientifically strict. The graduates acknowledged that after getting a job, they evaluate teachers more accurately and logically in comparison to their studying period. We recommended graduates’ viewpoints as one of the main components of faculty evaluation. For this purpose, it is better to set up a system of communication with graduates in the faculty evaluation unit. Considering the national nature of organizational structure of faculty members’ evaluation system, it may be useful to reestablish the methods and instruction of evaluation system and carry out it in universities.

## Data Availability

The datasets supporting the conclusions of this article are included within the article.

## Abbreviations

TBZMED: Tabriz University of Medical Sciences
P: Participant
